# Brain organoids engineered to give rise to glia and neural networks after 90 days in culture exhibit human-specific proteoforms

**DOI:** 10.3389/fncel.2024.1383688

**Published:** 2024-05-09

**Authors:** Tyler J. Wenzel, Darrell D. Mousseau

**Affiliations:** Cell Signalling Laboratory, Department of Psychiatry, University of Saskatchewan, Saskatoon, SK, Canada

**Keywords:** protein processing, heterogeneity, human brain, mouse brain, species differences, astrocyte, microglia, oligodendrocytes

## Abstract

Human brain organoids are emerging as translationally relevant models for the study of human brain health and disease. However, it remains to be shown whether human-specific protein processing is conserved in human brain organoids. Herein, we demonstrate that cell fate and composition of unguided brain organoids are dictated by culture conditions during embryoid body formation, and that culture conditions at this stage can be optimized to result in the presence of glia-associated proteins and neural network activity as early as three-months *in vitro.* Under these optimized conditions, unguided brain organoids generated from induced pluripotent stem cells (iPSCs) derived from male–female siblings are similar in growth rate, size, and total protein content, and exhibit minimal batch-to-batch variability in cell composition and metabolism. A comparison of neuronal, microglial, and macroglial (astrocyte and oligodendrocyte) markers reveals that profiles in these brain organoids are more similar to autopsied human cortical and cerebellar profiles than to those in mouse cortical samples, providing the first demonstration that human-specific protein processing is largely conserved in unguided brain organoids. Thus, our organoid protocol provides four major cell types that appear to process proteins in a manner very similar to the human brain, and they do so in half the time required by other protocols. This unique copy of the human brain and basic characteristics lay the foundation for future studies aiming to investigate human brain-specific protein patterning (e.g., isoforms, splice variants) as well as modulate glial and neuronal processes in an *in situ*-like environment.

## Highlights

- Initial steps of unguided brain organoids (BOs) formation dictate cell fate and composition.
- Optimizing unguided BO protocols halves the time needed for glia and mature neural networks to arise.
- BOs display human-specific protein banding patterns.

## 1 Introduction

Human brain organoids (BOs) were first described over 15 years ago (Eiraku et al., 2008), but there is still a limited understanding about which features of the human brain these cultures mimic. It is clear that BOs will continue to provide insight into human brain health and disease and reduce the use of animals in research. Yet, it is also clear that benchmarking human BOs, where ethically possible, to human brain tissue will be critical because species-specific protein processing often complicates the interpretation of data and its translation to the clinic (Webster et al., 1995; Bellinger et al., 2011; Jensen and Little, 2023; Maksour and Ooi, 2023; Wenzel et al., 2023a). Furthermore, the quality control of commercially available antibodies often relies on recombinant proteins or immortalized cell lines as positive controls; this is particularly important given that the protein banding pattern in a simplified system does not properly reflect the endogenous banding pattern in complex tissues such as brain parenchymal tissues and BOs, both of which have more multicellular machinery that could influence protein signatures (Haytural et al., 2019; Rosell et al., 2020). Key evidence to validate BOs as a model would be to consistently generate BOs with a cell composition similar to the human brain and demonstrate that BOs exhibit protein banding patterns similar to human parenchymal tissue.

BOs are not often used for immunoblotting experiments (Zhao et al., 2022). In part, this may be because stringent protocols without the use of concentrated proteinaceous capsules are required for replicable results (Hernández et al., 2022; Wenzel et al., 2023a). As such, it is unknown whether the proteins of BOs and human brain parenchyma are processed in a similar manner. We only identify two studies that have compared these human tissues with the mouse parenchyma (Lancaster et al., 2013; Sriram et al., 2020), and these studies only investigated a few proteins of interest. Currently, a majority of the BO literature uses immunofluorescence microscopy and RNA sequencing, which provide information on protein localization and levels of RNA transcripts, but does not provide information on protein processing (e.g., splice variants, post-translational modifications, cleavage) and does not accurately inform on protein levels unless volumetric analyses are conducted (Wenzel et al., 2023a,c). Thus, a wide comparison of cell and synapse markers between primary parenchymal tissues and BOs using immunoblotting would be beneficial, as it is the first step to determine whether BOs process proteins in a human-specific manner (Kroon et al., 2022).

To the best of our knowledge, there are no studies that determine whether human BOs process proteins in a human brain-specific manner, nor are there any studies that have identified culture parameters that affect the trajectory of BO maturation. Thus, it is uncertain whether BOs can be used to study protein processing, and direct comparison between BO studies may not be adequate due to differences in experimental conditions, such as composition of cell culture medium, that can influence the trajectory of BO maturation. Herein, we address these knowledge gaps by comparing the profiles of select proteins in human BOs, to profiles in human cortical (Ctx) and cerebellar (Cb) autopsy brain tissues and mouse Ctx tissues. We demonstrate that banding pattern and protein proportions of several proteins in human BOs are more similar to human Ctx or Cb tissues than mouse Ctx tissues, indicating that the proteins in human BOs are processed in a manner similar to the proteins in the human brain parenchyma. Furthermore, we generated BOs from the induced pluripotent stem cells (iPSCs) of female–male sibling donors. We chose this approach as it has been shown that familial cell lines result in more similar organoids (Jourdon et al., 2023). Our data corroborate these reported data as we observed limited within-donor variability between batches of human BOs. Furthermore, these *in vitro* cultures display less protein variability between samples than the human and mouse parenchymal tissues. Importantly, we also show that the first 24 h of BO culture is a critical window for maturation purposes. In summary, we demonstrate that human BOs exhibit human brain-like properties, and propose that these stem cell-based cultures are strong candidates for helping to bridge the gap between preclinical and clinical studies.

## 2 Materials and methods

### 2.1 Human and mouse tissues

Human autopsy tissues were obtained from the Douglas-Bell Canada Brain Bank (McGill University, Canada) and covered by the University of Saskatchewan’s Research Ethics Office Certificate of Approval ‘Bio 06–124’ (principal investigator: DM). Human donor information is described in Table 1.

**Table 1:** Donor information.

Human cortex (from neurocognitively normal donors)			
Donor sex	Donor age	Apolipoprotein E alleles	Cause of death
Male	61 years old	ε3/ε3	Pancreatic metastasis
	77 years old	ε3/ε3	Information not available
	60 years old	ε3/ε3	No discernable cause
Female	59 years old	ε3/ε3	Chronic obstructive pulmonary disease
	75 years old	ε3/ε3	Pulmonary edema
	91 years old	ε3/ε3	Information not available

C57Bl/6 J mouse cortex		
Donor sex	Donor age	Number of other mice in cage
Male	Postnatal week 11	1
	Postnatal week 14	1
	Postnatal week 15	3
Female	Postnatal week 11	4
	Postnatal week 14	2
	Postnatal week 15	2

Animal tissues were harvested by protocols approved by the University of Saskatchewan’s Animal Research Ethics Board (Animal Use Protocol No. 20060070; principal investigator: DM) and in accordance with the Canadian Council on Animal Care standards. C57Bl/6 J mice (Strain No. 000664) were received from the Jackson Laboratory (Bar Harbor, Maine, United States), housed in individually ventilated maintenance cages in a specific-pathogen free environment. Mice had free access to standard chow and water in a temperature-controlled room (21–22°C) with reverse lighting cycle (12 h dark/light). Mice (3 male and 3 female) were anesthetized by CO_2_ gas and then euthanized by decapitation at the age described in Table 1. Brain tissues were collected and the cortical region was isolated for protein extraction, and immunoblotting. In accordance with the 3Rs (replace, reduce, reuse) of animal research, we note that these treatment-naïve mouse brains were harvested during the course of another study.

### 2.2 Antibodies and reagents

L-ascorbic acid (LAA), ethylenediaminetetraacetic acid (EDTA), heparin, laminin from Engelbreth-Holm-Swarm murine sarcoma, Lowry assay kit (Peterson’s modification), poly-D-lysine hydrobromide, protease inhibitor cocktail (Cat#P8340), sodium selenite, and Terg-A-Zyme detergent were purchased from Millipore Sigma (Oakville, ON, Canada). Transforming growth factor-β1 (TGF-β1; cat #100–21) and fibroblast growth factor 2 (FGF2; cat#100-18B) were purchased from Peprotech (Cranbury, NJ, United States) was purchased from Peprotech (Cranbury, NJ, United States). Recombinant human transferrin (cat#777TRF029) was purchased from InVitria (Fort Collins, CO, United States). Radioimmunoprecipitation assay (RIPA) 10x buffer and the ROCK inhibitor Y-27632 (cat# 13624S) were purchased from Cell Signaling Technologies (Whitby, ON, Canada). BrainPhys™ Neuronal Medium, EasySep™ Release Human CD45 Positive Selection Kit, and the STEMdiff™ Cerebral Organoid Kit (cat#08570) were obtained from STEMCELL Technologies (Vancouver, BC, Canada). A full list of antibodies and their suppliers is provided in Table 2. All other reagents were sourced from Fisher Scientific (Ottawa, ON, Canada).

**Table 2:** List of antibodies and reagents used.

Target	Catalogue number	Dilution for blotting (IB) and immunohistochemistry (IHC)	Amount of protein loaded for IB
Primary antibodies			
Rabbit anti-β-actin	Cell signaling technology Cat#4970	1:5,000 (IB)	Figure 3: 15 μg; Figure 7: 10 μg; Supplementary Figure S1: 10 μg
Rabbit anti-GAPDH	Cell signaling technology cat#5174	1:2000 (IB)	Figure 3: 15 μg; Figure 7: 10 μg
mouse anti-GFAP	Millipore sigma cat#3893	1:1,000 (IB) 1:200 (IHC)	Figure 6: 10 μg; Supplementary Figure S1: 10 μg
Rabbit anti-IBA1	Abcam cat# ab178846	1:1,000 (IB)	Figure 5: 10 μg
Rabbit anti-OLIG2	Millipore sigma cat#AB9610	1:1,000 (IB) 1:200 (IHC)	Figure 6: 15 μg; Supplementary Figure S1: 10 μg
Rabbit anti-P2RY12	Atlas antibodies cat#HPA014518	1:1,000 (IB)	Figure 5: 20 μg
Mouse anti-NeuN	Millipore sigma cat#MAB377	1:2,000 (IB)	Figure 4: 10 μg
Rabbit anti-TLR4	Santa cruz biotechnology cat#sc-293072	1:1,000 (IB)	Figure 7: 10 μg
Mouse anti-TMEM119	Biolegend cat#A16075D	1:500 (IB) 1:200 (IHC)	Figure 5: 20 μg; Supplementary Figure S1: 10 μg
Rabbit anti-TUBB3	Millipore sigma cat#T2200	1:5,000 (IB) 1:200 (IHC)	Figure 3: 15 μg; Figure 4: 10 μg; Supplementary Figure S1: 10 μg
Rabbit anti-SYN1	Cell signaling technology Cat#5297	1:1000 (IB)	Figure 4: 10 μg
Chicken anti-MAP2	Abcam cat#ab5392	1:200 (IHC)	N/A
Mouse anti-S100B	Millipore sigma cat#AMAb91038	1:200 (IHC)	N/A
Rabbit anti-GFAP	Cell signaling technology cat#12389S	1:200 (IHC)	N/A
Rabbit anti-PU.1	Cell signaling technology cat#2258S	1:200 (IHC)	N/A
Mouse anti-MBP	Cell signaling technology cat#83683S	1:1000 (IB) 1:200 (IHC)	Figure 6: 15 μg
Mouse anti-CNP	Millipore sigma cat#MAB326	1:200 (IHC)	N/A
Secondary antibodies			
IRDye® 680RD anti-rabbit IgG	LI-COR biosciences cat#926–68,071	1:20,000 (IB)	N/A
IRDye® 680RD anti-mouse IgG	LI-COR biosciences cat#926–68,070	1:20,000 (IB)	N/A
IRDye® 800CW anti-rabbit IgG	LI-COR biosciences cat#926–32,211	1:20,000 (IB)	N/A
IRDye® 800CW anti-mouse IgG	LI-COR biosciences cat#926–32,210	1:20,000 (IB)	N/A
Donkey anti-rabbit IgG, Alexa Fluor™ 594	Thermofisher scientific cat#A21207	1:2000 (IHC)	N/A
Goat anti-Mouse IgG, Alexa Fluor™ 488	Thermofisher Scientific cat#A11029	1:2000 (IHC)	N/A

TUBB3, βIII-Tubulin; CNP, 2′,3′-cyclic nucleotide 3′ phosphodiesterase; GFAP, glial fibrillary acidic protein; GAPDH, glyceraldehyde 3-phosphate dehydrogenase; IgG, immunoglobulin G; IBA1, ionized calcium-binding adapter molecule 1; NeuN, neuronal nuclei; OLIG2, oligodendrocyte transcription factor 2; MAP2, microtubule-associated protein 2; MBP, myelin basic protein; P2RY12, purinergic receptor P2Y12; S100B, S100 calcium-binding protein B; PU.1, spi-1 proto-oncogene; SYN1, synapsin 1; TLR4, toll-like receptor 4; TMEM119, transmembrane protein 119.

### 2.3 Inducible pluripotent stem cell (iPSC) maintenance

UCSD086i-6-3 (86i, male) and UCSD087i-6-4 (87i, female) iPSC lines, which are derived from a male–female sibling pair, were purchased from WiCell (Madison, WI, United States) and confirmed to be karyotypically normal by the provider. These were used in all experiments and were cultured in feeder-free conditions on 6-well tissue culture plates coated with Matrigel™, human embryonic stem cell (hESC)-qualified matrix (Corning™ 354277), or Geltrex™ hESC-qualified matrix and maintained at 37°C in humidified 5% CO_2_ and 95% air atmosphere. iPSCs were maintained in embryoid body (EB) formation media 3 with 25 ng/mL FGF2 and 1 ng/ml TGF-β1.

### 2.4 Generation of unguided brain organoids

Human unguided BOs were generated as described elsewhere (Lancaster and Knoblich, 2014; Wenzel et al., 2023b). Briefly, iPSCs were incubated with 0.5 mM EDTA for 5 min. They were removed from the plate with a cell scraper, centrifuged, and resuspended at 9 × 10^4^ iPSCs/ml in EB formation media (Table 3) with 10 μM Y-27632 inhibitor. ~9,000 iPSCs were seeded in a 96-well ultra-low attachment round-bottom plate. 24 h later (day 1), 100 μL of EB formation medium 3 (see Table 3) were added to each well. On day 4, using a 200 μL wide-bore pipette tip and ensuring minimal transfer of EB formation medium, individual EBs were transferred to separate wells of a 24-well ultra-low attachment plate containing 500 μL of induction media. On day 7, media was replaced with 500 μL of ice-cold expansion media and on day 10, 1 mL of maturation media was added to each well and incubated until day 17 at 37°C in humidified 5% CO_2_ and 95% air atmosphere on an orbital plate shaker set at 0.118 *g*. Once a week, media was replaced with 750 μL of maturation media, followed by an additional 500 μL being added 4 days later. BOs in culture were harvested at day 90 and protein extracts were prepared for standard denaturing SDS-PAGE immunoblotting, while BO sections were fixed for immunofluorescence microscopy. Brightfield images of BOs were taken with an Olympus CK53 microscope. The induction, expansion, and maturation media used were from the STEMdiff™ Cerebral Organoid Kit purchased from STEMCELL Technologies. We confirmed these kits use high levels of heparin.

**Table 3:** Formulations of EB formation media tested.

EB formation media 1	EB formation media 2 (Lancaster and Knoblich, 2014)	EB formation media 3	EB formation media 4	EB formation media 5
EB formation medium from STEMdiff™ cerebral organoid kit	DMEM-F12 with HEPES	DMEM-F12 with HEPES	DMEM-F12 with HEPES	DMEM-F12 with HEPES
	1X GlutaMAX™	2 mM L-alanyl-L-glutamine dipeptide^b^	2 mM L-alanyl-L-glutamine dipeptide^b^	2 mM L-alanyl-L-glutamine dipeptide^b^
	3% v/v embryonic stem cell-qualified fetal bovine serum	20 μg/mL recombinant human insulin^a^	20 μg/mL recombinant human insulin^a^	20 μg/mL recombinant human insulin^a^
		20 μg/mL recombinant human transferrin^a^	20 μg/mL recombinant human transferrin^a^	20 μg/mL recombinant human transferrin^a^
		20 ng/mL sodium selenite^a^	20 ng/mL sodium selenite^a^	20 ng/mL sodium selenite^a^
	20% v/v knockout replacement serum	0.2 mg/mL L-ascorbic acid^a^	0.2 mg/mL L-ascorbic acid^a^	0.2 mg/mL L-ascorbic acid^a^
	1X minimal essential medium-non-essential amino acids			
	0.0007% v/v 2-mercaptoethanol			
	4 ng/mL FGF2		4 ng/mL FGF2	25 ng/mL FGF2

a In subsequent experiments, we have observed a range of concentrations for these reagents create viable EBs, although they cannot be absent from the EB FM. These concentrations were chosen as a trade-off between cost and growth rate of EBs.

b L-alanyl-L-glutamine dipeptide can be replaced by L-glutamine. Although, it should be noted that the stability of L-glutamine is affected by light exposure, pH and temperature.

HEPES, 2-[4-(2-hydroxyethyl)piperazin-1-yl]ethanesulfonic acid; FGF2, basic fibroblast growth factor; DMEM-F12, dulbecco’s modified eagle medium with nutrient mixture F-12; EB, embryoid body.

### 2.5 Immunoblotting and immunofluorescence

For immunoblotting, five BOs were pooled and homogenized in RIPA buffer containing protease inhibitor cocktail. Samples were triturated with a one-ml pipette, disrupted using a 22-gauge needle, and homogenized using sonication. Protein concentration of samples was quantified using the Lowry assay and normalized to 0.5 μg/μl in 1% loading buffer (0.2 M TRIS pH 6.8, 40% glycerol, 8% sodium dodecyl sulfate, 20% β-mercaptoethanol, 0.4% bromophenol blue). Samples were not heated so as to avoid any protein aggregation. Resolved proteins (10–20 μg per lane, as indicated in Table 2) were electroblotted onto a nitrocellulose membrane and blocked in 5% bovine serum albumin (BSA) in TRIS-buffered saline (TBS: 25 mM Tris pH 7.4, 137 mM NaCl) for 1 h. Membranes were then washed and incubated overnight at 4°C with primary antibodies (Table 2) diluted in 5% BSA in TBS-T (TBS with 0.1% Tween®20). After three washes with TBS-T over 30 min, secondary fluorophore-labelled antibodies (Table 2) were added for 1 h, followed by three washes. Proteins were visualized with a LI-COR Odyssey® Imager and analyzed with manufacturer’s software (Image Studio 5.3.5, LI-COR Biosciences, Lincoln, NE, United States).

For immunofluorescence microscopy, BOs were transferred to a 15 mL conical tube, washed three times with sterile PBS, fixed in 4% paraformaldehyde for 16 h at 4°C, and then washed three times with 0.1% Tween®20 in PBS (PBS-T). 24 h later, the fixed organoids were transferred to 15% sucrose in PBS for 24 h, followed by an additional 24 h incubation in 30% sucrose in PBS. Organoids were transferred to a mould and embedded in optimal cutting temperature (OCT) compound. Tissues were quickly frozen and sectioned (15 μm) using a Leica CM1950 cryostat and microtome, and then mounted on SuperFrost™ Plus microscope slides. Organoid sections were outlined with a Pap pen, washed thrice with PBS at room temperature for 15 min to remove OCT, immersed in blocking solution (5% normal donkey serum in PBS-T) for 1 h, and incubated for 16 h at room temperature with primary antibodies (Table 2) diluted in 5% BSA in PBS-T. After six washes with PBS-T over 1 h, organoid sections were incubated with secondary antibodies (Table 2) at room temperature in a humidified chamber for 2 h, followed by three additional washes. ProLong™ Glass Antifade Mountant with NucBlue™ was added and a coverslip placed on top. Slides were cured for 24 h at room temperature prior to imaging on a Zeiss AxioImager M.1 widefield microscope or an Olympus FV-1000 confocal microscope.

### 2.6 Multielectrode array recording

A pilot study was done to determine whether our brain organoids were functionally active. BOs were plated on a MaxOne high-density multiectrode array (MaxWell Biosystems, Zurich, Switzerland) following manufacturer’s instructions. In brief, electrodes were treated with 1% (w/v) Terg-A-Zyme for 2 h. Next, electrodes were coated with 0.1 mg/mL poly-D-lysine dissolved in a borate buffer for 1 h, washed three times, and then coated with 0.04 mg/mL laminin dissolved in culture media for 1 h. BOs were then allowed to attach to the multielectrode array over 2 weeks in a CO_2_ incubator set to 37°C. Media was replaced twice a week with 600 μL of BrainPhys™ Neuronal Medium. After a two-week incubation, neural network activity was measured using the Activity Scan Assay followed by the Network Activity Assay in the MaxLab Live software (MaxWell Biosystems).

### 2.7 Cell viability

The PrestoBlue assay was used (following manufacturer’s instructions) to measure the reduction of resazurin to resorufin (a red fluorescent compound) by living cells in organoids. Data are presented as fluorescence signal of resorufin.

### 2.8 Data analysis

GraphPad Prism 9.2 software was used for all statistical analyses. Data [mean ± standard deviation (SD)] were analyzed using the non-parametric (1) two-tailed Mann–Whitney test, or the (2) Kruskal-Wallis test, followed by the Dunn’s or the Sidak’s post-hoc test. Significance was established at *p* < 0.05. For mouse and human parenchyma data, each data-point (i.e.*, n* value) was derived from a different donor (Table 1). For BO data, the individual data-points (i.e.*, n* value) were derived from batches of organoids grown on different days.

## 3 Results

### 3.1 Culture conditions during embryoid body (EB) formation dictate yield and protein composition of brain organoids

**Figure 1:**
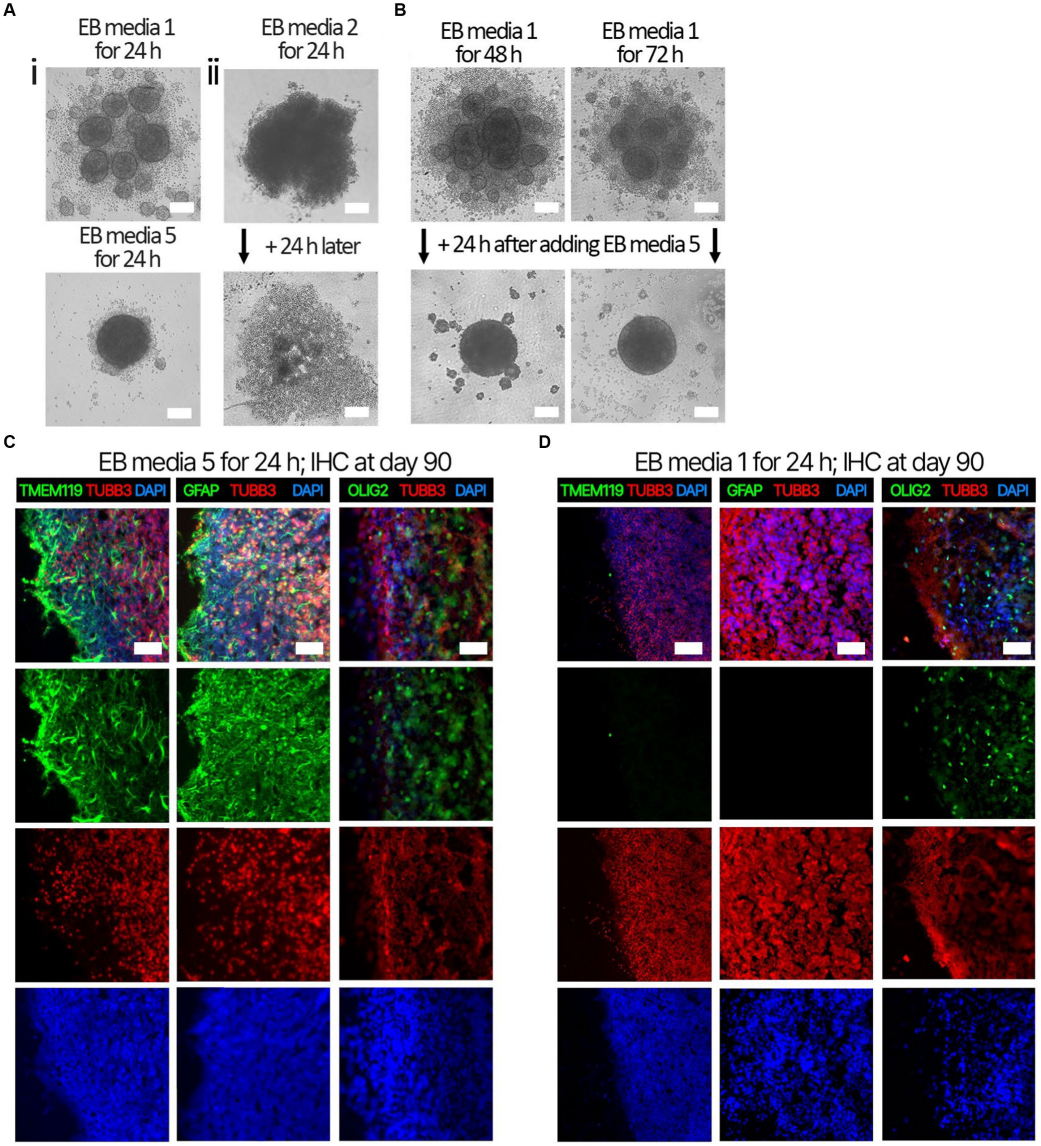
Representative images of EB formation data in Table 4. iPSCs from the same cell suspension were seeded in either (**Ai,** *top*) EB formation media 1, (**Ai,** *bottom*) EB formation media 5, or (**Aii**) EB formation media 2, which is media described in Lancaster and Knoblich (2014). (**Ai**, *top*) After 24 h, EB formation media 1 always formed multiple, small, EBs inadequate for BO generation, whereas *(bottom)* EB formation media 5 could form the single, large EB required for BO generation. **(Aii)** After 24 h, EB formation media 2 produced an aggregate with undefined borders, which disaggregated into a monolayer of single cells by 48 h. **(B)** Inadequate EBs formed by EB formation media 1 were repaired within 24 h post-exposure to EB FM 5. **(C)** Higher levels of TMEM119, GFAP and OLIG2 were detected at day 90 in BOs exposed to EB formation media 5 for the first 24 h of generation **(D)** compared to BOs exposed to EB formation media 1 for the first 24 h. **(C,D)** Image colors are enhanced by linear increases to gain to improve visualization, with any change in gain or contrast applied to all images in a series. Scale bars: **(A,B)** 100 μm and **(C,D)** 100–200 μm. Sections were counter-stained with DAPI (nuclear stain: blue).

Several EB formation media (Table 3) were used to test the hypothesis that early culture parameters dictate BO yield and the trajectory of BO maturation. We first compared the efficacy of different EB formation media (Table 3) to generate single, large EBs in the first 24 h, which is a necessary result for the subsequent maturation of BOs. Media included a commercial kit (EB formation media 1), the formulation described in Lancaster and Knoblich (2014) (EB formation media 2), and a chemically-defined media formulation with different concentrations of FGF2 (EB formation media 3–5). EB formation media 5 yielded significantly more (e.g., 96%) viable EBs than any of the other formulations tested (e.g., 0–25%) (Table 4). Experiments were repeated 8–16 times on different days. EB formation media 1 formed multiple inadequate EBs (Figure 1Ai, *top*), while EB formation media 3–5 more often formed the expected single, round EB (Figure 1Ai, *bottom*). In all attempts, EB formation media 2 did not form stable EBs, as they lost structural integrity within 48 h (Figure 1Aii). Interestingly, we observed that EB formation media 5 induced the multiple inadequate EBs generated by EB formation media 1 to aggregate into a single, round EB (Figure 1B). Supplementary Figure S1 shows that the reaggregated BOs expressed TUBB3, TMEM119, GFAP, and OLIG2 at levels similar to those in Figure 1D at *in vitro* day 90 according to immunoblotting.

**Table 4:** Comparing embryoid body yield of different formation media.

EB formation media used for first 24 h	EB formation media used from 24–96 h	% Viable EBs at 96 h (viable EBs/total EBs)
EB formation media 1 (Commercial kit)	EB formation media 3	10.1% (99/984)
EB formation media 2 (Most cited)		0% (0/192)
EB formation media 3		11.5% (12/104)
EB formation media 4		25% (32/128)
EB formation media 5		96.8% (422/436)

**Figure 2:**
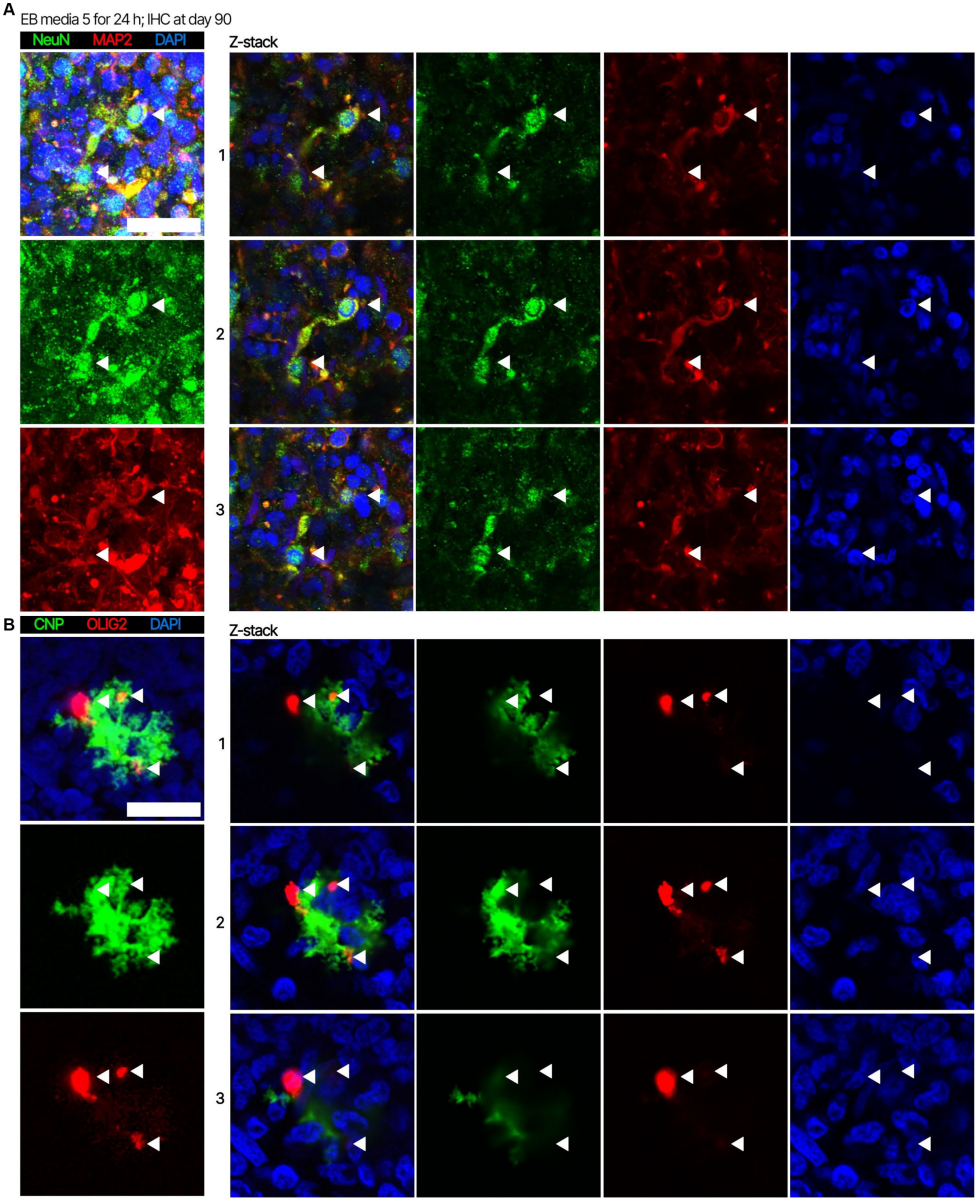

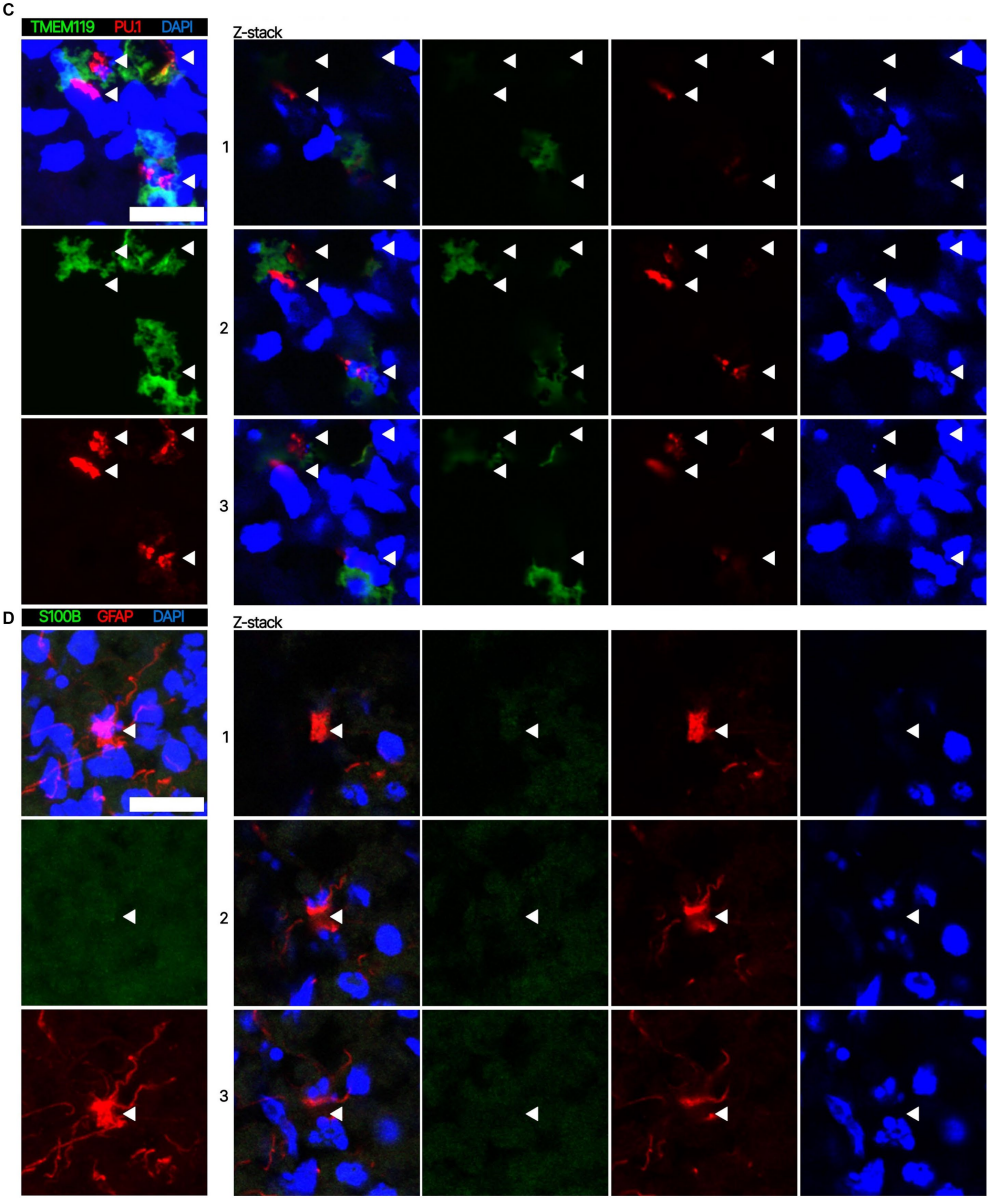
Representative high magnification images of the cells in BOs exposed to EB formation media 5 for the first 24 h of generation. Z-stacked images of BO sections stained for **(A)** neuronal markers NeuN and MAP2, **(B)** oligodendrocytes markers CNPase and OLIG2, **(C)** microglial markers TMEM119 and PU.1, and **(D)** astrocytic markers GFAP and S100B at *in vitro* day 90. (**A–D**, *left*) Projection images of maximum signal intensity from Z-stacked images are shown, as are (*right*) images of optical slices that are 2 μm apart. **(A-D)** Arrows mark the same location on images in a series to aid visualization. Scale bars: 20 μm. In all cases, sections were counter-stained with DAPI (nuclear stain: blue).

Next. we assessed whether the BOs generated with high yields affected the maturation of BOs, as we hypothesized that the higher yields indicate these cultures would have healthier cells to facilitate their maturation. Thus, we investigated brain cell types present at day 90 in BOs generated with EB formation media 5 for the first 24 h (Figure 1C) or EB formation media 1 for the first 24 h (Figure 1D). Immunohistochemistry shows BOs exposed to EB formation media 5 for 24 h expressed GFAP and TMEM119 at day 90 (Figure 1C), unlike BOs exposed to EB formation media 1 (Figure 1D). Additionally, BOs expressing GFAP and TMEM119 expressed more OLIG2 (Figure 1C) than BOs that did not (Figure 1D). Z-stacked images show the co-localization of neuronal markers NeuN and MAP2 (Figure 2A), oligodendrocytes markers CNPase and OLIG2 (Figure 2B), as well as microglial markers TMEM119 and PU.1 (Figure 2C). Figure 2D shows the astrocytic marker GFAP stains a star-shaped cell, but the protein S100B, which is sometimes considered an astrocytic marker, does not co-localize with GFAP.

### 3.2 Optimized culture conditions reproducibly generate functional brain organoids with similar metabolism and protein levels

**Figure 3:**
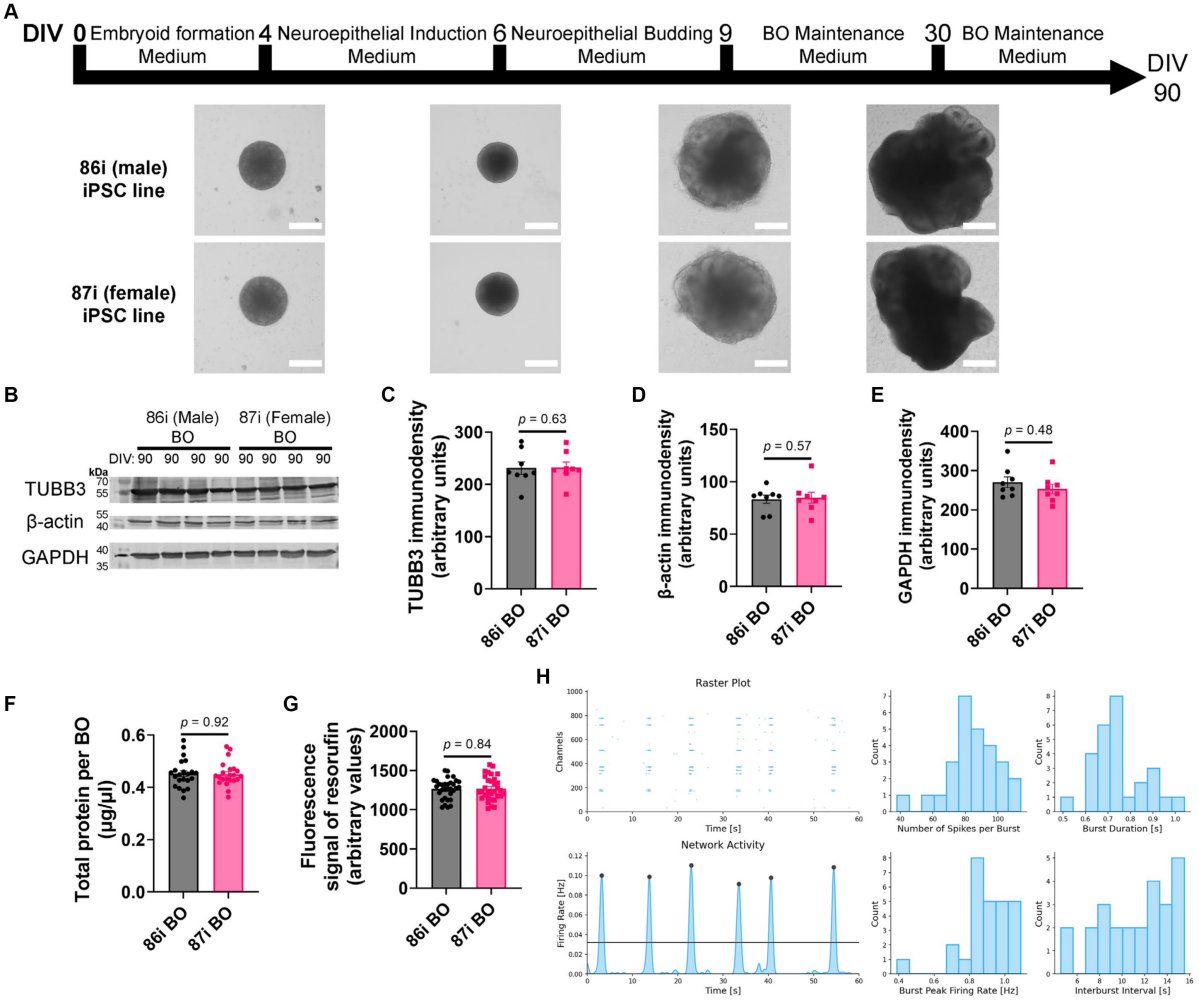
BOs were grown as described in Wenzel et al. (2023b) using EB formation media 5 for 24 h. **(A)** BOs derived from 86i and 87i cell lines exhibit all visual markers of proper development. **(B–E)** At day 90, the levels of housekeeping proteins TUBB3 (*p* = 0.63*, U* = 27), β-actin (*p* = 0.57, *U* = 26), and GAPDH (*p* = 0.48, *U* = 24) are consistently expressed at similar levels, **(F)** as are the levels of total protein (*p* = 0.92, *U* = 237.5), indicating these cultures grow to similar sizes and complexities at similar rates. **(G)** The ability of BOs to metabolize resazurin into resorufin is also similar at day 90 (*p* = 0.84, U = 496), indicating similar metabolic activity. **(C–G)** Data (means ± SD) analyzed according to the two-tailed Mann–Whitney test. **(B–E)** BO data were derived by pooling five organoids. The five pooled BOs were from the same batch, but each sample (i.e., lane or data-point) was from a different batch of five organoids (batches defined as BOs generated on different days from iPSCs of a different vial). **(F,G)** Total protein and resorufin data-points represent individual organoids. **(H)** Two weeks after plating on a multielectrode array, the instrument detects neural network activity in 86i (male) BOs cultured for 90 days. (*left*) Spontaneous action potentials (spikes) and network burst electrical activity were observed, and (*right*) the electrophysiological properties of this network activity are summarized.

Next, we hypothesized that greater cellular complexity of BOs would result in the earlier appearance of brain-like characteristics in a reproducible manner. We confirmed this using 86i (male) and 87i (female) familial iPSC lines and BOs generated using EB formation media 5 for the first 24 h. Figure 3A shows that the male and female BOs displayed all the expected visual characteristics of normal unguided BO development, as defined elsewhere (Lancaster and Knoblich, 2014), which are indicative of brain-like layering. At day 90, BOs consistently expressed comparable levels of housekeeping proteins TUBB3, β-actin, and GAPDH regardless of the batch of iPSCs used to generate the BOs (Figures 3B–E). The volume of organoids as measured by total protein (Figure 3F) as well as their ability to metabolically reduce resazurin (Figure 3G) were also similar between batches. We also plated BOs on a multielectrode array to test whether there was any detectable neural activity at day 90. Two weeks after plating the organoids, we detected spontaneous and synchronized network electrical activity (Figure 3H). Figure 3 demonstrates BOs grown using EB formation media 5 for the first 24 h meet all the visual and functional characteristics of a BO, and thus this media formulation was used for the remaining experiments.

### 3.3 Comparison of banding patterns for select proteins in brain organoids to human and mouse brain parenchymal tissues

**Figure 4:**
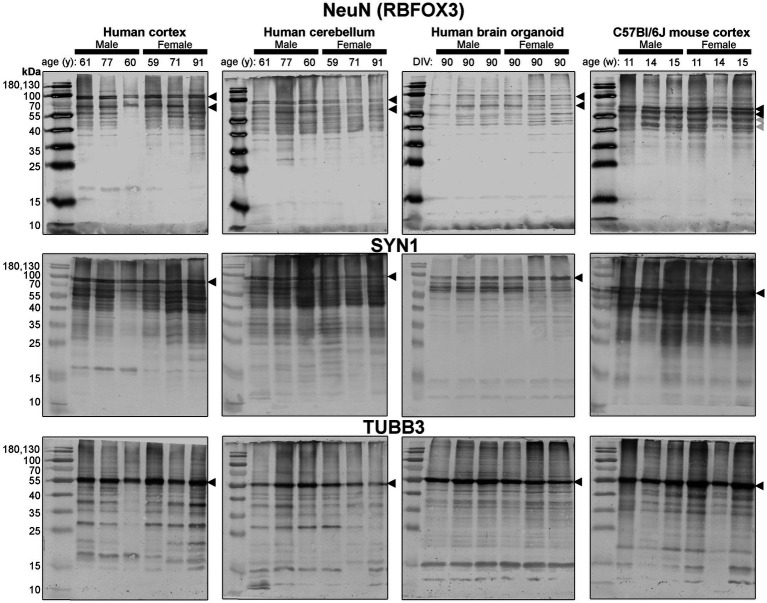
Immunoblots of neuron-associated proteins NeuN (*top*), SYN1 (*middle*) and TUBB3 (*bottom*). Protein homogenates for primary mouse and human tissues were derived from different donors (Table 1), while BO homogenate was derived by pooling five organoids. The five BOs were from the same batch, but each sample (i.e., lane) was from a different batch of five organoids (batches defined as BOs generated on different days from iPSCs of a different vial). Samples of human Ctx and Cb, BOs as well as mouse Ctx were simultaneously run on an 12% SDS-PAGE gel, transferred onto a nitrocellulose membrane, and treated with antibodies in the same containers. The amount of protein loaded for each sample and the antibodies used are indicated in Table 2. Membranes images of the same protein were also taken at the same time as a single image, and only cropped and digitally repositioned for publication. No post-processing was done on any image, and so the immunodensity of bands are visually comparable from one blot to the next. We note that TUBB3 was probed overtop of the IBA1 immunoblot shown in Figure 5, but the 55 kDa band was clearly the only new band detected by TUBB3 antibodies. Arrowheads indicate the specific bands used for estimations of protein expression for Figures 8, 9. DIV, days in vitro, w, week; y, year; NeuN, neuronal nuclei; TUBB3, β3-tubulin; SYN1, synapsin 1.

**Figure 5:**
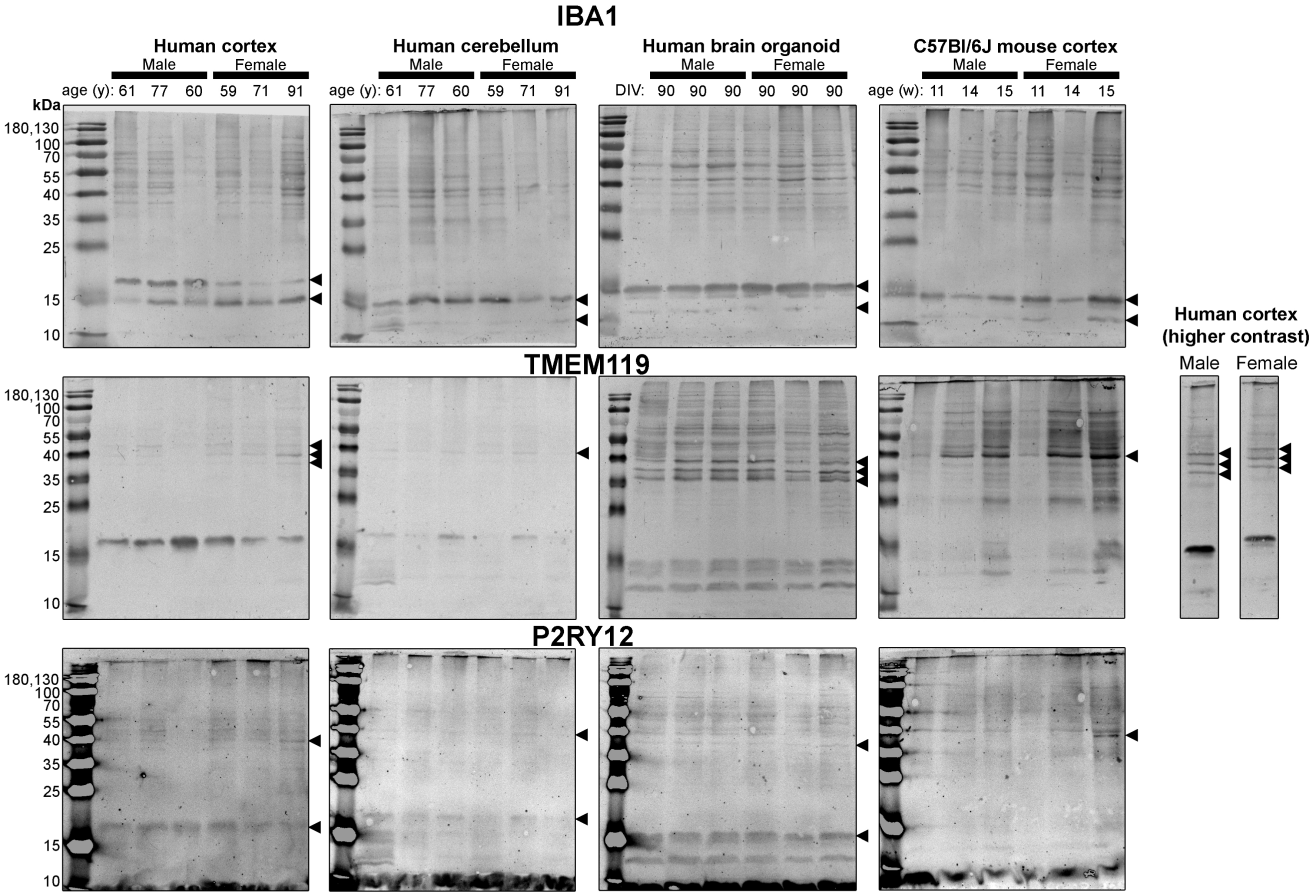
Immunoblots of microglia-associated proteins IBA1 (*top*), TMEM119 (*middle*) and P2RY12 (*bottom*). Samples are the same protein homogenates described in Figure 4, and the individual antibodies were used to probe all membranes on the same day so that the immunodensity of bands are visually comparable across blots. Arrowheads indicate the specific bands used for estimations of protein expression for Figures 8, 9. DIV, days in vitro; w, week; y, year; IBA1, ionized calcium-binding adapter molecule 1; TMEM119, transmembrane protein 119; P2RY12, purinergic receptor P2Y12.

**Figure 6:**
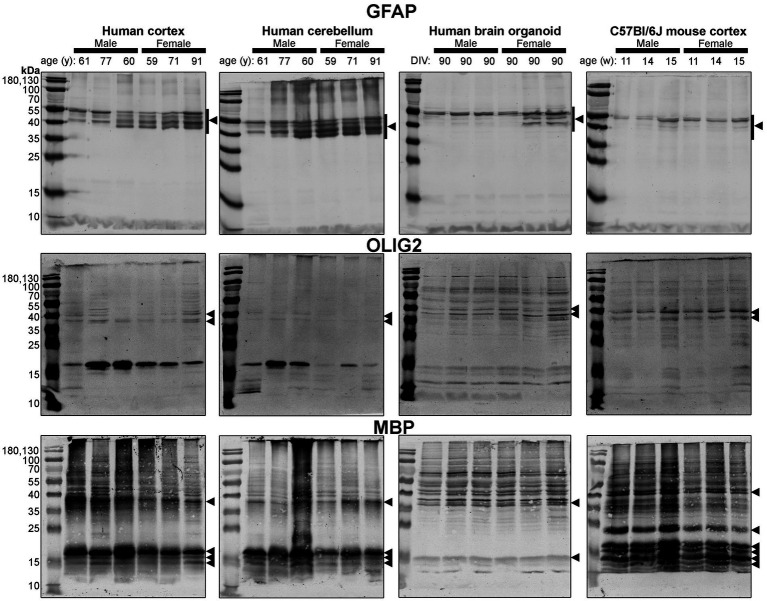
Immunoblots of macroglia (astrocyte and oligodendrocyte)-associated proteins GFAP (*top*), OLIG2 (*middle*), and MBP (*bottom*). Samples are the same protein homogenates described in Figure 4, and are probed by the individual antibodies simultaneously to allow for proper comparison of samples between blots. Arrowheads indicate the specific bands used for estimations of protein expression for Figures 8, 9. DIV, days in vitro; w, week; y, year; GFAP, glial fibrillary acidic protein; MBP, myelin basic protein, OLIG2, oligodendrocyte transcription factor 2.

**Figure 7:**
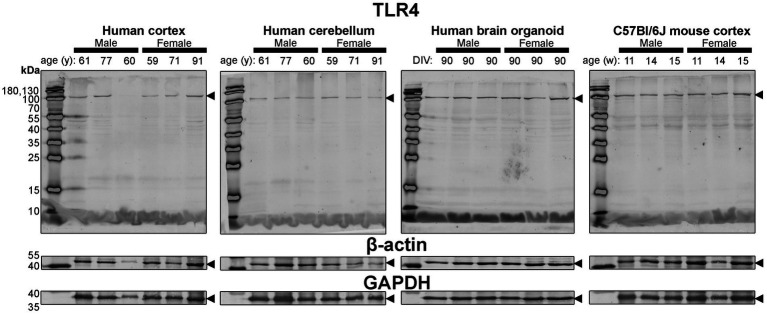
Immunoblots of TLR4 (*top*) as well as housekeeping proteins β-actin (*middle*) and GAPDH (*bottom*). Samples are the same protein homogenates described in Figure 4, and are blotted simultaneously to allow for proper comparative densitometric analysis across samples. β-actin and GAPDH blots are cropped as only one band was detected. Arrowheads indicate the specific bands used for estimations of protein expression for Figures 8, 9. DIV, days in vitro; w, week; y, year; TLR4, toll-like receptor 4; GAPDH, glyceraldehyde-3-phosphate dehydrogenase.

Since these BOs exhibit the electrophysiological characteristics of brain tissue, as well as more cellular complexity than other BO cultures, we hypothesized that these BOs would also have the cellular machinery to process proteins in a manner similar to the human brain parenchyma. So, we compared the protein banding patterns of human BOs to human and mouse parenchymal tissue to test our hypothesis. This experiment fills a critical knowledge gap, as there is a paucity of studies that have directly compared the immunoblotting results of human BOs to human and mouse parenchymal tissue; thus, it is unclear how similar the human and mouse brain are from a protein processing level, despite the many reports implicating interspecies differences in signalling pathways (Mestas and Hughes, 2004; Smith and Dragunow, 2014; Monaco et al., 2015; Sun et al., 2016; Fischer, 2021). In particular, we compared BOs at 90 days in culture to human Ctx and Cb tissues and mouse Ctx tissues in case there were brain region-specific protein banding patterns. In order to avoid bias, we include full immunoblots (rather than cropped images) from each of our experiments (Figures 4–7). These clearly demonstrate that BOs exhibit protein banding patterns that are more similar to the human Ctx or Cb tissue than to the mouse Ctx tissue. Immunoblots were scanned at the same time with the same imaging settings, so the banding intensity is comparable visually.

First, we immunoblotted for neuron-associated proteins (Figure 4). In BOs, the NeuN signal included bands at 55, ~70, and ~ 100 kDa, which were very similar to the patterns in the human Ctx and Cb samples. In contrast, NeuN was immunodetected primarily as a ~ 55–60 kDa doublet in mouse tissues (Figure 4, *top*). Similar band patterns were also observed for SYN1 in human brain samples and BOs, where SYN1 migrated primarily as a 55 kDa doublet in mouse tissues (Figure 4, *middle*). TUBB3 exhibits a similar protein banding pattern between all tissues tested (Figure 4, *bottom*).

Next, we probed for proteins associated with microglia, the resident immune cells of the brain (Figure 5). IBA1 (a marker of microglial state) was detected as 12 kDa and 15 kDa species in all tissues analyzed, except for the human Ctx, which expresses the 15 kDa species and a species at ~17 kDa (Figure 5, *top*). TMEM119 (a marker of homeostatic microglia) was detected primarily as a ~ 17 kDa species in human Ctx, with a lesser triplet migrating between 45 and 55 kDa (Figure 5, *middle*). In contrast, the triplet was the primary signal in BOs, although it migrated between 35 and 43 kDa in this tissue, and a smaller species (<15 kDa) was also detected. In mouse, TMEM119 was detected primarily as a 43-kDa species (Figure 5, *middle*). The expression of P2RY12 (a marker of one of the fundamental signaling systems in microglia) was weak in all tissues tested, but migrated at the expected size, e.g., ~40 kDa; however, we observed a proportionally stronger signal at ~18 kDa in all three human tissues, which was not observed in the mouse (Figure 5, *bottom*).

Different isoforms of GFAP (a marker of astrocytes) migrating between 35 and 55 kDa (Kamphuis et al., 2012, 2014) were observed in the human tissues, with a tendency for more signal in the female samples (Figure 6, *top*). In contrast, only one isoform was observed in mouse Ctx tissues (Figure 6, *top*). OLIG2 (a marker of oligodendrocytes) was detected as two bands ~40 kDa in all samples, albeit at weaker intensity in the human Cb samples (Figure 6, *middle*). We note a 17 kDa band that appears in OLIG2 blots of human parenchymal tissues, with a strong detection in the male samples. MBP (a marker of myelinating oligodendrocytes) was detected predominately as a ~ 18.5 kDa protein in human brain parenchyma samples. We also detected the ~37, 17.2 and 14 kDa splice variants in the human brain tissues, but not any 21.5 kDa protein. We primarily detected ~37 kDa and 14 kDa bands in brain organoids. In contrast, the mouse cortex expresses protein splice variants, such as the 21.5, 18.5, 17.2, 14 kDa isoforms, more uniformly, and the larger protein migrated at ~40 kDa.

Finally, expression of TLR4—an activator of immune (microglial) cells that is reported to be differentially expressed between humans and mice (Smith and Dragunow, 2014)—and levels of the housekeeping proteins, β-actin and GAPDH, were essentially similar across all tissues (Figure 4).

### 3.4 Differences and similarities of select protein levels in brain organoids as well as in human and mouse brain parenchymal tissues

**Figure 8:**
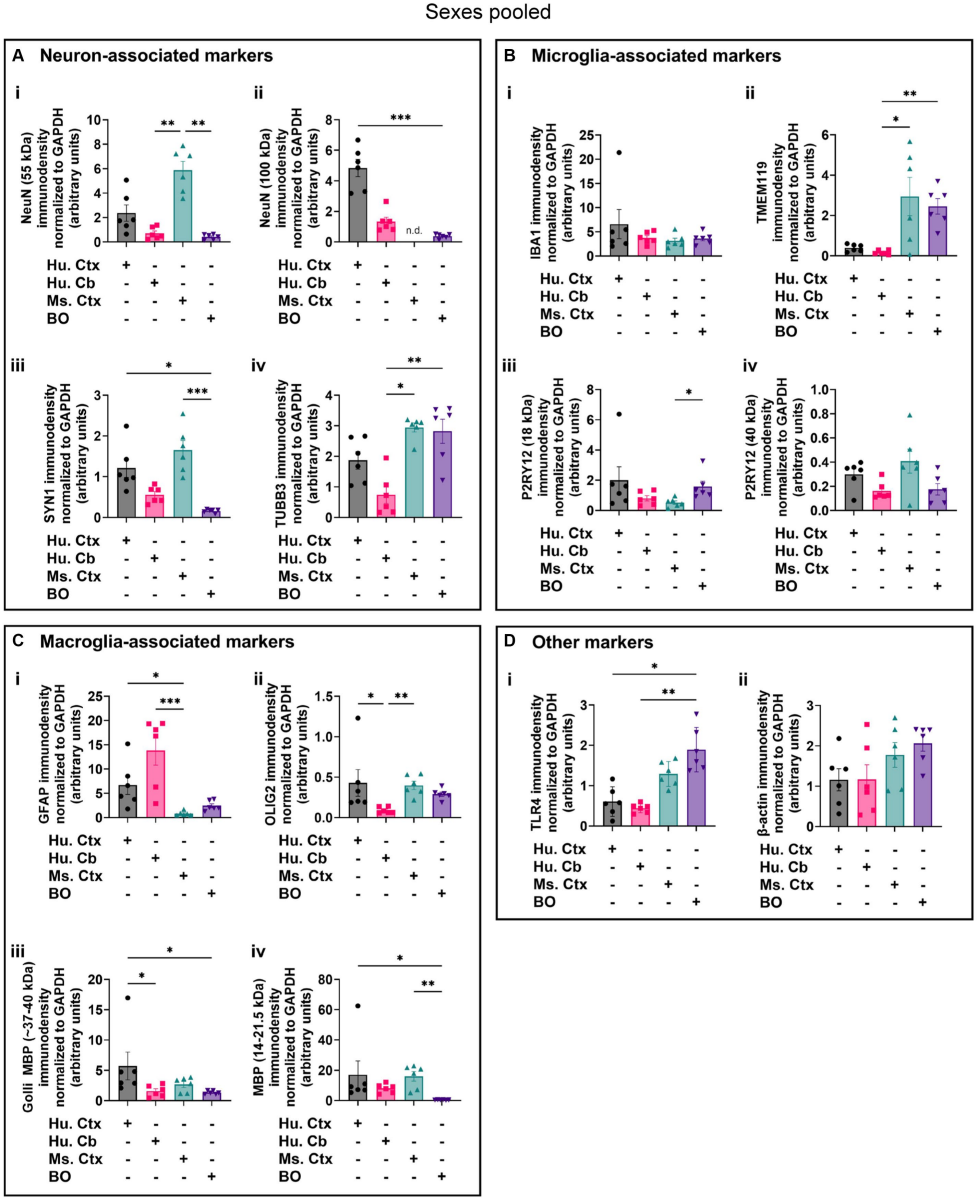
Densitometry of immunoblots (*n* = 3 males +3 females, pooled) shown in Figures 4–7 comparing the expression levels of **(A)** neuron-associated markers (i, NeuN-55 kDa, *p = 0.0004*, *H* = 17.97; ii, NeuN-100 kDa, *p < 0.0004*1, *H* = 15.16; iii, SYN1, *p = 0.0002*, *H* = 19.21; iv, TUBB3, *p = 0.0018*, *H* = 15.03;), **(B)** microglia-associated markers (i, IBA1, *p = 0.72*, *H* = 1.31; ii, TMEM119, *p = 0.0033*, *H* = 13.70; iii, P2RY12–18 kDa, *p = 0.023*, *H* = 8.57; iv, P2RY12–40 kDa, *p = 0.064*, *H* = 7.25), **(C)** macroglia (astrocyte and oligodendrocyte)-associated markers (i, GFAP, *p = 0.0005*, *H* = 17.55; ii, OLIG2, *p = 0.0023*, *H* = 14.54; iii, Golli MBP, *p = 0.0092, H* = 11.53; iv, MBP, *p = 0029, H* = 14.01), and **(D)** other proteins of interest (i, TLR4, *p = 0.0006*, *H* = 17.46; ii, β-actin, *p = 0.13*, *H* = 5.57). Data presented as means ± SD and normalized to the housekeeping protein GAPDH. Data-points from primary mouse and human tissues were derived from different donors (Table 1), while individual BO data-points were derived by pooling five organoids. The five BOs pooled per sample were from the same batch, but individual samples (i.e., data-point) represent a different batch of five organoids (batches defined as BOs generated on different days from iPSCs of a different vial). ^*^p < 0.05, ^**^p < 0.01, and ^***^p < 0.0001 according to the Dunn’s *post hoc* test and *p* and H value according of Kruskal-Wallis test.

Densitometry of immunoblot shown in Figures 4–7 were conducted and summarized in Figure 8. When pooling the data points by biological sex, we demonstrate that the protein immunodensity of NeuN (55 kDa), TUBB3 (Figure 8A), IBA1, TMEM119, P2RY12 (40 kDa and 18 kDa) (Figure 8B), GFAP, OLIG2 (Figure 8C), and β-actin (Figure 8Dii) are similar between BOs and human Ctx tissues. The immunodensity of TMEM119 (Figure 8Bii) and TLR4 (Figure 8Di) are more similar between BOs and mouse Ctx tissues, as the human Ctx and Cb homogenates have comparatively less TMEM119 and TLR4. In contrast, we also noted some dissimilarities between BOs and the other tissues. For example, the human Ctx has the highest levels of the 100 kDa NeuN species (Figure 8Ai) as well as Golli MBP (~37–40 kDa) (Figure 8Ciii) compared to other tissue tested, and human and mouse Ctx tissues have higher levels of SYN1 (Figure 8Aiii) and MBP (14-21.5 kDa) (Figure 8Civ) than BOs. We also observed that the mouse Ctx has significantly lower levels of GFAP than the human parenchymal tissues but at levels similar to BOs, and OLIG2 immunodensity is lower in the human Cb compared to the human and mouse Ctx (Figure 8C).

### 3.5 Sex-dependent differences of select proteins in brain organoids as well as human and mouse parenchymal tissues

**Figure 9:**
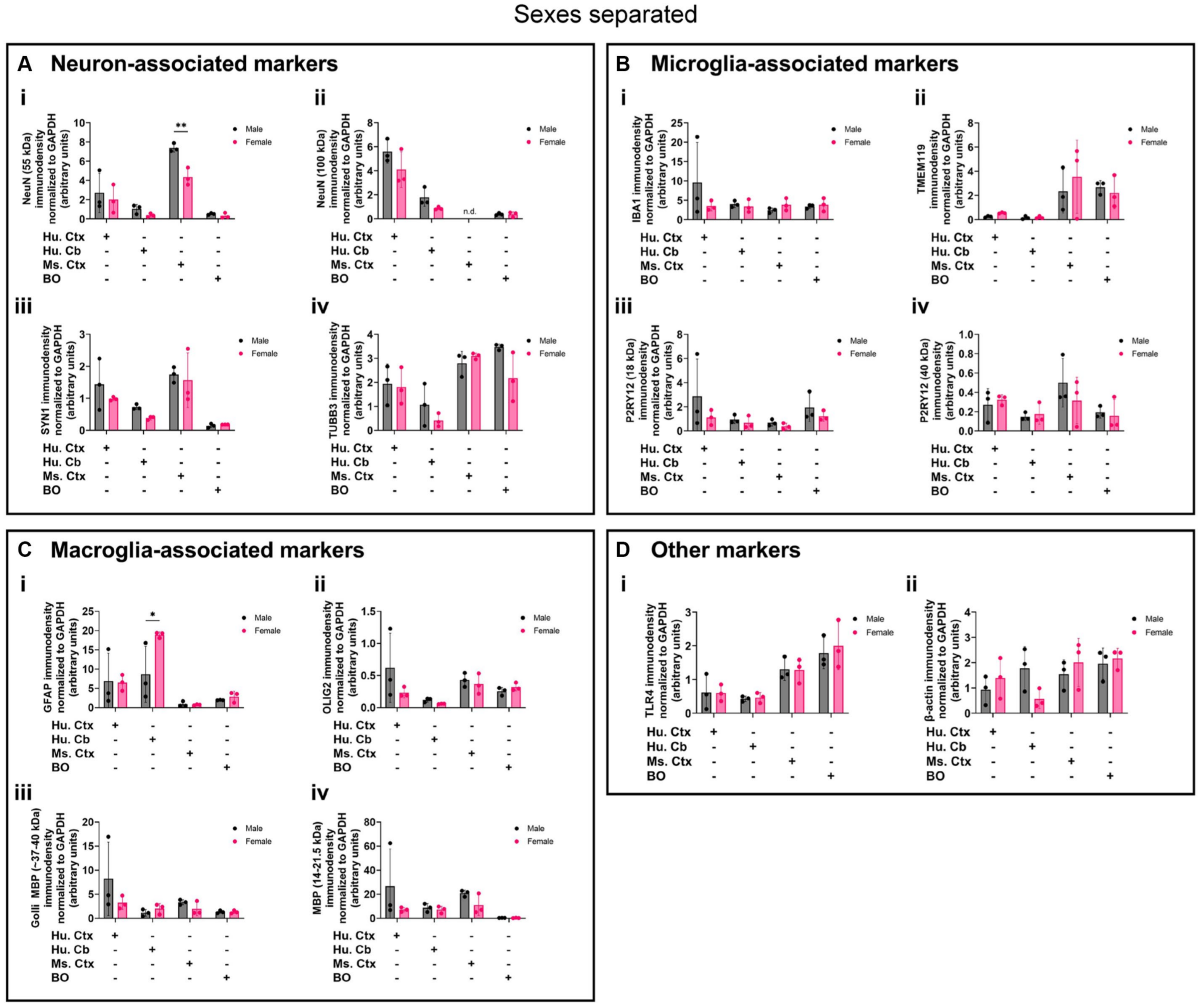
Separation of data-points shown in Figure 8 by biological sex to compare sex-dependent expression levels of **(A)** neuron-associated markers (i, NeuN-55 kDa; ii, NeuN-100 kDa; iii, SYN1; iv, TUBB3), **(B)** microglia-associated markers (i, IBA1; ii, TMEM119; iii, P2RY12–18 kDa; iv, P2RY12–40 kDa), **(C)** macroglia (astrocyte and oligodendrocyte)-associated markers (i, GFAP; ii, OLIG2; iii, Golli MBP; iv, MBP), and **(D)** other proteins of interest (i, TLR4; ii, β-actin). Data presented as means ± SD and normalized to the housekeeping protein GAPDH. Data-points from primary mouse and human tissues were derived from different donors (Table 1), while BO data-points were derived by pooling five organoids from different batches (batches defined as BOs generated on different days from iPSCs of a different vial). ^*^p < 0.05 and ^**^p < 0.01 according to the Sidak’s *post hoc* test.

Biological sex can influence the macrostructure of the human parenchyma (DeCasien et al., 2022), but we could not identify any studies that investigated sex differences in cell markers in BOs or brain parenchyma. Given this, it is unclear whether there exist differences in the density of cell types in BOs or human and mouse brain parenchymal tissues. Thus, we re-analyzed the data shown in Figure 8 to determine if any sex differences exist in our samples. The 55-kDa NeuN band intensity was significantly less in female mouse Ctx tissue than male mice (Figure 9Ai), and GFAP immunodensity was significantly higher in female human Cb tissues than male Cb tissues (Figure 9Ci). No sex-specific differences were observed for any of the other proteins measured.

## 4 Discussion

BOs are emerging as a relevant model of human brain health and disease, but the extent to which they model the human brain parenchyma is still largely uncertain. A majority of studies compare the transcriptome of BOs to those of adult and fetal parenchymal tissues and demonstrate these cultures display human brain-like layers (Lancaster et al., 2013; Velasco et al., 2019). However, the transcriptome does not always align with the proteome and seldom considers splice variants (Gonzàlez-Porta et al., 2013; Bathke et al., 2019). Post-translational modifications are also rarely considered, even though such modifications can underpin causative or adaptive mechanisms in health and disease, responses to stimuli, or simply aid in regulating gene expression and maintaining cellular homeostasis. Thus, and in support of this emerging field, we feel it is critical to understand how well BOs recapitulate protein expression patterns observed in human brain and which of the protocols for generating BOs are the most likely to yield translational outcomes. There has been a widespread shift towards using regionalized BOs, but the lack of cell diversity arising from these methods are a concern (Del Dosso et al., 2020). For example, many BOs only express synaptic and glial proteins after 6 months in culture (Velasco et al., 2019; Sivitilli et al., 2020). It is plausible that each protocol exhibits advantages and demerits, and the choice of the BO model could vary based on the research question. Herein, we demonstrate (1) the culture conditions in the first 24 h of unguided BO development dictate cell fate and composition, and (2) that our unguided human BOs express proteins in a human brain-specific manner.

Several methods for incorporating microglia-like cells into BOs have been developed (Abud et al., 2017; Ormel et al., 2018; Bodnar et al., 2021; Xu et al., 2021; Cakir et al., 2022; Sun et al., 2022), and rely on co-culturing organoids with iPSC monolayer-derived microglia or hematopoietic cells, doxycycline-inducible genes, or specific nutrient cocktails. The protocol we describe herein is most similar to the protocol described in Ormel et al. (2018) and Bodnar et al. (2021); however, our protocol can generate BOs that contain innate microglia-like cells, even with standard high levels of heparin. Notably, our observed low levels of IBA1 and high levels of TMEM119 are indicative of homeostatic microglia, and thus this protein signature represents an appropriate baseline to study neuroinflammation (Lier et al., 2021).

Furthermore, we show that small, multiple EBs may arise in cultures due to a nutrient deficiency, and not due to unhealthy iPSCs as suggested elsewhere (Lancaster and Knoblich, 2014). Our protocol has been optimized to the point that it allows for the rescue of these small, multiple EBs and induces them to aggregate by simply replenishing nutrients. These observations indicate that there is much yet to be developed in terms of the optimal BO protocol, and while we are certainly well along that path there will likely be more bioengineering studies needed to fully appreciate all the factors that influence cell fate as well as EB yields.

Studies have shown the variability in cell composition of BOs is predominately driven by the genetic differences of iPSCs from different families (Jourdon et al., 2023); using iPSCs from a highly-characterized familial stem cell collection (Panopoulos et al., 2017), we corroborate this by showing minimal batch-to-batch variability —as measured by several different assays, including total protein, resazurin reduction, and expression of housekeeping proteins— between and within our familial (brother and sister) iPSC lines. This consistency occurs without compromising the original visual developmental hallmarks of BOs indicative of brain-like layering (Lancaster and Knoblich, 2014). It is essential that studies are able to consistently replicate the protein composition of BOs, and the types of cells that arise in these cultures, as inconsistent composition of cultures will likely bias data interpretation (Wenzel et al., 2023a) and as shown elsewhere, studies reporting BO phenotype variability are the same studies that also struggle to generate BOs with consistent protein profiles (Hernández et al., 2022). Stringent standard operating procedures, such as using the same lot number of commercial culture supplements (particularly those supplements that are widely recognized for having batch-to-batch differences), may be essential to reproducibly generate BOs (Chen et al., 2008; van der Valk et al., 2010; Sloan et al., 2018).

Interestingly, the protein profile of our BOs has similar, and possibly exhibit even less, between-donor variability than parenchymal tissues from human donors or clonal mice, as evidenced, for example, by proteins that are in continual flux such as IBA1. We detected similar isoforms of GFAP in our BOs and some of our human donors, and observed species differences between our human BO and brain parenchymal tissues and the mouse Ctx tissues, which expressed fewer isoforms (Kamphuis et al., 2012, 2014). It will be important to extend these studies to include BOs from additional donors to determine whether BOs retain the donor heterogeneity that we observe in human brain samples. The transcriptomic study by Jourdon et al. (2023) indicates that at least some donor heterogeneity is retained in BO cultures.

There is limited comparison of protein expression patterns (based on immunoblotting) in human and mouse brain tissues (Ishii et al., 2009; Bayés et al., 2012), complicating the interpretation of immunoblots (Rosell et al., 2020). For example, a NeuN doublet migrating just above 55 kDa has been observed in unguided BOs after 90 days *in vitro* (DIV) and an undisclosed section of adult human and mouse brain parenchyma (Sriram et al., 2020); this differs from the molecular weight of the doublet we observed in human tissues. Young et al. (2021), using anti-TMEM119 antibodies, detected a triplet between 38 and 70 kDa in mouse brain parenchyma when 15 μg of homogenate was loaded, and only detected a single, 50 kDa band when 3 μg was loaded (Young et al., 2021). In contrast, we observed a triplet with human tissues (20 μg) probed for TMEM119, but only detected a single band in mouse Ctx tissues. It is unclear whether these additional bands at unexpected molecular weights are post-translational modifications, splice variants, or non-specific binding, as surmised elsewhere (Rosell et al., 2020). In the specific case of MBP, the bands in the human and mouse brain parenchyma resolve at the molecular weights of known splice variants (Staugaitis et al., 1990; Boggs, 2006); although, we do not detect any species at 21.5 kDa in the human brain parenchyma. The 14-kDa MBP detected in BOs is one of the two species that form compact myelin sheaths. The ~37–40 kDa bands may represent Golli MBP (Siu et al., 2015). Golli MBP is very understudied, but its expression in brain organoids enables the investigation of many important lines of inquiry, as Golli MBP is implicated in myelination events in aging and remyelination events in disease (Paez et al., 2012). It is also possible that certain bands, such as the 17 kDa bands we observed with anti-OLIG2 and anti-TMEM119 antibodies, are breakdown products as suggested in other studies (Haytural et al., 2019). Regardless of whether the protein bands detected in our study are proteoforms or non-specific binding, our data indicate that BOs are a human-like tissue as they demonstrate protein banding more similar to human brain parenchymal tissue, as evidenced most strikingly by the NeuN, SYN1 and TMEM119 blots.

While there are well-documented sex differences in regional volume in brain, the cytoarchitectural basis for these differences is unclear (DeCasien et al., 2022). For example, sex-dependent abundance of brain cells such as microglia in the mouse have been reported (Bordt et al., 2020), whereas consistent cell proportions have been reported for human brain parenchymal tissue (Johansen et al., 2022). Elsewhere, sex-dependent brain volume differences have been attributed to changes in cell type proportion as a function of age (Chen et al., 2023). Our data largely support studies demonstrating a lack of generalized sex-dependent differences in human brain cell proportions (DeCasien et al., 2022; Johansen et al., 2022), and we further show that BOs themselves do not seem to exhibit sex differences in protein composition at 90 DIV. Interestingly, we do see a sex difference in the levels of NeuN in mouse Ctx tissue, but no differences in the density of microglia in these same tissues, which contrasts the reports included in the Bordt et al. (2020) review. We also observe a sex-dependent difference in GFAP immunodensity in human Cb tissues, but the underlying basis for this difference is unclear. For instance, mouse models of traumatic brain injury indicate that GFAP levels are modulated in a sex-dependent manner, suggesting that external factors may contribute to any observed sex-dependent differences in GFAP (Wright et al., 2017; Sass et al., 2021).

Our preliminary investigation did detect spontaneous and synchronized neural network activity, thus corroborating other studies using BOs (Fair et al., 2020; Sharf et al., 2022; Van Lent et al., 2023). Unguided BOs have been shown to exert synchronized activity between five to six months in culture (Fair et al., 2020; Sharf et al., 2022), although one study could not detect any such spontaneous synchronicity without the presence of microglia-like cells (Popova et al., 2021). Since microglia-like cells facilitate the maturation of neural networks and other cell types in BOs (Popova et al., 2021; Park et al., 2023), it is possible the innate microglia-like cells in our BOs are underpinning our ability to detect synchronized neural network activity as early as three months in culture. In general, electrophysiological parameters of unguided BOs are inconsistent between studies (Fair et al., 2020; Popova et al., 2021; Sharf et al., 2022), with Fair et al. (2020) detecting spontaneous burst activity every 25 s, whereas activity was observed every five seconds or every 125 s in Sharf et al. (2022) and Popova et al. (2021), respectively. BOs generated using alternative protocols also exhibited a slow firing rate, with burst activity being detected every 80 s (Van Lent et al., 2023). The electrophysiology parameters of our BOs were similar to those reported by Fair et al. (2020) and Sharf et al. (2022), as we detected burst activity approximately every 12 s. We continue to investigate how innate microglia affect electrophysiological functioning in our BOs.

In summary, we demonstrated that human BOs display protein banding and proportions more similar to human Ctx or Cb tissues than mouse Ctx tissues. This is the first time that BOs have been shown to largely process and express proteins in a human brain-specific manner. This strongly suggests BOs may be very well suited for the study of human brain health and pathology, and could extend into the study of the processes that underlie some of this protein banding, such as alternative mRNA splicing and post-translational modifications that may, or may not, have analogous counterparts in mouse models. Technical limitations prohibit commonly used techniques in BO studies, such as microscopy and RNA sequencing, from readily answering these same research questions. We also clearly demonstrate that early BO culture conditions dictate cell fate and composition and support the growing evidence that stem cell-based experiments should use familial iPSC lines where possible to mitigate any variability as a function of genetic background. Importantly, much earlier expression of neural network activity as well as astrocytes, oligodendrocytes, microglia in our BOs may have widespread benefit to investigations into neuron–glia interactions and communications. Our future studies will investigate which cellular signalling pathways are conserved within BOs, and given the presence of the innate microglia in our BOs, translational studies on neuroinflammation in health and disease could more readily be considered.

## Data availability statement

The original contributions presented in the study are included in the article/Supplementary material, further inquiries can be directed to the corresponding author.

## Ethics statement

The studies involving humans were approved by University of Saskatchewan’s Research Ethics Office Certificate of Approval ‘Bio 06–124’ (principal investigator: DM). The studies were conducted in accordance with the local legislation and institutional requirements. The human samples used in this study were acquired from the Douglas-Bell Canada Brain Bank (McGill University, Canada). Written informed consent for participation was not required from the participants or the participants’ legal guardians/next of kin in accordance with the national legislation and institutional requirements. The animal study was approved by University of Saskatchewan’s Animal Research Ethics Board (Animal Use Protocol No. 20060070; principal investigator: DM). The study was conducted in accordance with the local legislation and institutional requirements.

## Author contributions

TW: Conceptualization, Data curation, Formal analysis, Funding acquisition, Investigation, Methodology, Writing – original draft, Writing – review & editing. DM: Conceptualization, Funding acquisition, Supervision, Writing – review & editing.

## Glossary

86i: UCSD086i-6-3 male cell line
87i: UCSD087i-6-4 female cell line
BO: brain organoid
BSA: bovine serum albumin
Cb: cerebellar
Ctx: cortical
DIV: days *in vitro*
EB: embryoid body
EDTA: ethylenediaminetetraacetic acid
FGF2: fibroblast growth factor 2
GAPDH: glyceraldehyde-3-phosphate dehydrogenase
GFAP: glial fibrillary acidic protein
hESC: human embryonic stem cell
IBA1: ionized calcium-binding adapter molecule 1
IFN-γ: interferon-γ
iPSC: induced pluripotent stem cell
LAA: L-ascorbic acid
NeuN: neuronal nuclei
OLIG2: oligodendrocyte transcription factor 2
P2RY12: purinergic receptor P2Y12
SD: standard deviation
SYN1: synapsin 1
TBS: TRIS-buffered saline
TGF-β1: transforming growth factor-β1
TLR4: toll-like receptor 4
TMEM119: transmembrane protein 119
TUBB3: β3-tubulin
w: week
y: year
