# Supplementary material for: Brain organoids engineered to give rise to glia and neural networks after 90 days in culture exhibit human-specific proteoforms
Source: Front Cell Neurosci. 2024 May 9;18:1383688. doi: 10.3389/fncel.2024.1383688 (PMC11111902; doi:10.3389/fncel.2024.1383688)
Supplement: Supplementary file 1 [file Data_Sheet_1.docx]

**Supplementary Figures and Legends**

**Supplementary Figure 1**
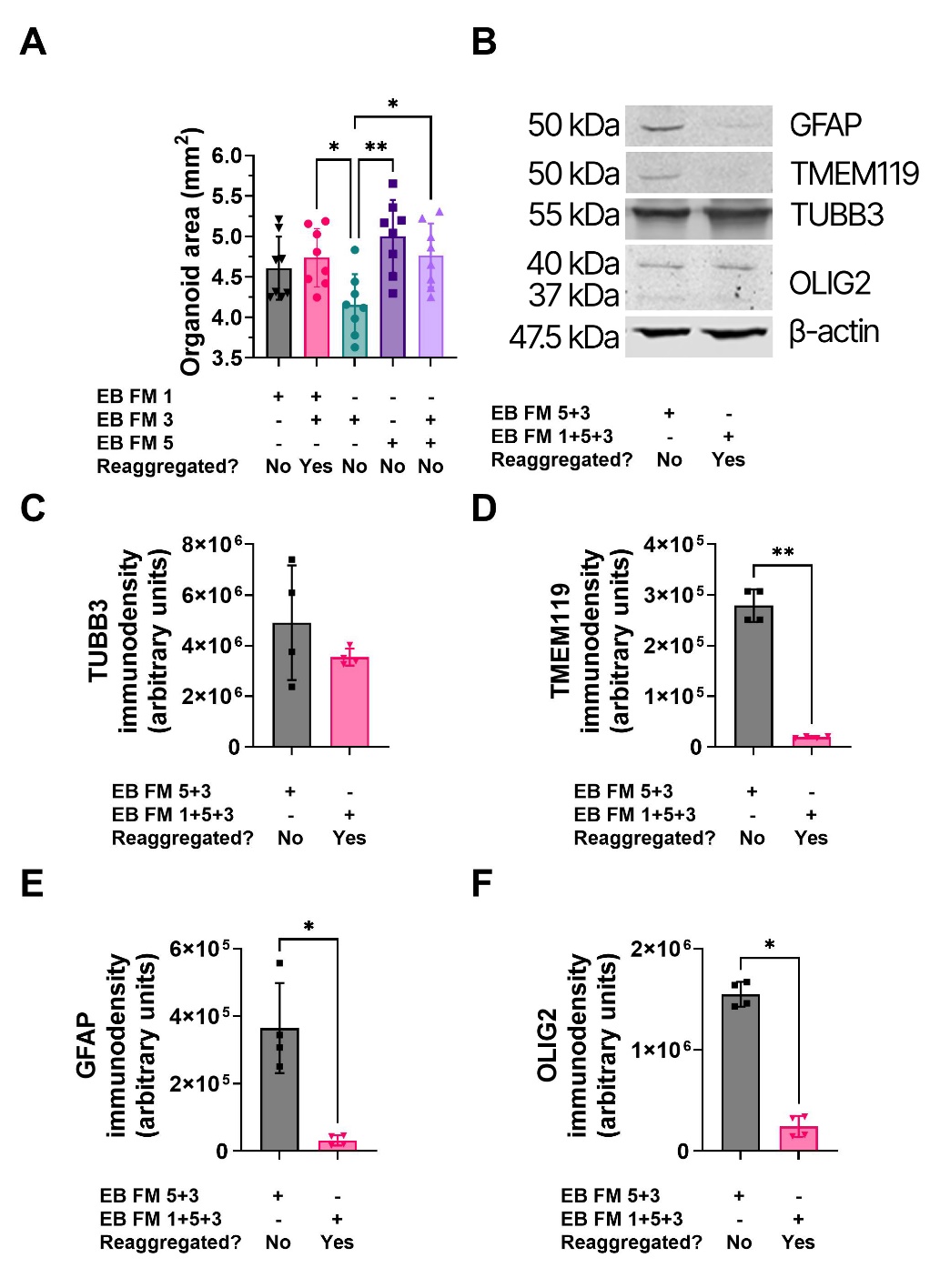


**Supplementary Figure 1: (A)** BOs generated with only EB FM 3 where significantly smaller at day 90 compared to a majority of the other EB FM combinations tested. **(B-F)** As we observed that EB FM 5 was able to induce the aggregation of multiple EBs, we tested whether reaggregation had any overt effect on protein density. **(B)** Representative immunoblots of samples derived from reaggregated BOs and those not needing reaggregation are shown. **(C)** TUBB3 levels were similar between the BOs, but **(D)** TMEM119, **(E)** GFAP, and **(F)** OLIG2 levels were lower, which is in-line with the representative microscopy images shown in **Figure 1.**
